# Valorizing date palm spikelets into activated carbon-derived composite for methyl orange adsorption: advancing circular bioeconomy in wastewater treatment—a comprehensive study on its equilibrium, kinetics, thermodynamics, and mechanisms

**DOI:** 10.1007/s11356-024-34581-3

**Published:** 2024-08-03

**Authors:** Mazen S. F. Al-Hazeef, Amel Aidi, Lynda Hecini, Ahmed I. Osman, Gamil Gamal Hasan, Mohammed Althamthami, Sabrina Ziad, Tarik Otmane, David W. Rooney

**Affiliations:** 1 Laboratory of LARGHYDE, University of Biskra, P.O. Box 145, 07000 Biskra, Algeria; 2 Department of Industrial Chemistry, University of Biskra, P.O. Box 145, 07000 Biskra, Algeria; 3 Scientific and Technical Research Center for Arid Zones CRSTRA, University of Biskra, PO Box 145, 07000 Biskra, Algeria; 4 Laboratory of LARHYSS, University of Biskra, BP 145 RP, 07000 Biskra, Algeria; 5 School of Chemistry and Chemical Engineering, Queen’s University Belfast, Belfast, Northern Ireland BT9 5AG UK; 6 Department of Process Engineering and Petrochemical, Faculty of Technology, University of El Oued, 39000 El Oued, Algeria

**Keywords:** Sustainable composite, Agricultural waste valorization, Azo dye adsorption, Water remediation, Bioresource utilization, Circular bioeconomy

## Abstract

Leveraging date palm spikelets (DPS) as a precursor, this study developed a DPS-derived composite (ZnO@DPS-AC) for water treatment, focusing on methyl orange (MO) removal. The composite was synthesized through ZnCl2 activation and pyrolysis at 600 °C. Comprehensive characterization was conducted using TGA, FTIR, XRD, SEM/EDS, and pH_PZC_. Characterization revealed a highly carbonaceous material (> 74% carbon) with significant porosity and surface functional groups. ZnO@DPS-AC demonstrated rapid MO removal, achieving over 45% reduction within 10 min and up to 99% efficiency under optimized conditions. The Langmuir model-calculated maximum adsorption capacity reached 226.81 mg/g at 20 °C. Adsorption mechanisms involved hydrogen bonding, π-π interactions, and pore filling. The composite showed effectiveness in treating real wastewater and removing other pollutants. This study highlights the potential of agricultural waste valorization in developing efficient, sustainable adsorbents for water remediation, contributing to circular bioeconomy principles.

**Supplementary Information:**

The online version contains supplementary material available at 10.1007/s11356-024-34581-3.

## Introduction

In an era where industrial and agricultural advancements have markedly contributed to environmental contamination, the pervasive dissemination of synthetic dyes poses a formidable challenge to both ecological integrity and public health (Ramutshatsha-Makhwedzha et al. 2022). These dyes, originating from industries such as food, pharmaceuticals, personal care, papermaking, painting, leather, and textiles, are difficult to degrade and pose severe threats to the environment and health (Farhan Hanafi and Sapawe 2020; Jiang et al. 2023; Sun et al. 2023). Annually, the global utilization of over 700,000 tons of dyes, with more than 11% inadvertently entering aquatic ecosystems, underscores the urgent need for effective remediation strategies (Wei et al. 2023). Dyes that are released into the environment without undergoing any treatment can result in various environmental effects. These include water and soil pollution, as well as health risks to humans, even at low concentrations (Shah et al. 2021; Sunthar et al. 2023).

Among these pollutants, methyl orange (MO), a widely used acidic anionic mono-azo dye, epitomizes the perils associated with dye effluents due to its recalcitrance and toxicological profile, threatening aquatic biota and human health alike (Ramath et al. 2023; Shah et al. 2021). Its low biodegradability and high water solubility make it persistent in water bodies (Shah et al. 2021), while its aromatic and –N = N– groups contribute to its toxicity (Loc et al. 2022). Exposure to MO can harm aquatic life and pose serious health threats to humans, including increased heart rate, vomiting, and even tissue necrosis (Azami et al. 2012; Gong et al. 2013).

Given the limitations of traditional wastewater treatment methodologies, including cost and secondary pollution issues, the exploration of adsorption techniques emerges as a vital alternative, offering simplicity, cost-efficiency, and high efficacy (Iwuozor et al. 2021). Those techniques include advanced oxidation processes, ion exchange, photocatalytic degradation, electrochemical degradation, ultrafiltration, coagulation-flocculation, and others (Islam et al. 2024).

Adsorption emerges as a paramount technique for the remediation of water contaminated with synthetic dyes such as MO, commended for its simplicity, cost-effectiveness, scalability, and exemplary removal efficiency (Msaadi et al. 2023; Pradhan et al. 2023). The quest for effective adsorbents has led to exploring diverse materials ranging from biopolymers, resins, nanoparticles, composites, clays, and, most notably, activated carbon. Among these, activated carbon and activated carbon-derived composites are distinguished by their expansive surface area, many functional groups, and environmental compatibility, making them the preferred choice for MO decontamination due to their superior adsorption capacity (De Smedt et al. 2024). Nevertheless, the conventional reliance on coal for activated carbon production poses significant sustainability and economic concerns, particularly in light of a market projected to burgeon to USD 7.0 billion by 2028, from an evaluation of USD 4.4 billion in 2023, with prices reaching up to USD 1700/ton in 2018 (De Smedt et al. 2024; Hock et al. 2018; Markets 2023). This scenario underscores the imperative for sustainable alternatives.

Within the framework of the circular bioeconomy (Mergbi et al. 2023), which emphasizes the valorization of waste materials and promotes resource sustainability, agricultural wastes have surfaced as a viable and ecologically responsible source of adsorbents, offering a dual benefit of pollutant mitigation and resource optimization. This economic model prioritizes the use of renewable resources and minimizes waste generation. By transforming leftover biomass materials into valuable products, like adsorbents for pollution cleanup, the circular bioeconomy embraces a zero-waste approach. This concept is seen as essential for transitioning to a more sustainable future. Following these principles, various agricultural residues, including Egyptian doum palm shells (Tcheka et al. 2024), pinewood (De Smedt et al. 2024), Neem chips (Sunthar et al. 2023), date seeds (Alardhi et al. 2023), coconut shells (Kamdod and Kumar 2022), pine cones (Kaya and Uzun 2021), pomelo peel (Zhang et al. 2020), sugarcane bagasse, orange peel, and spent coffee grounds (Martínez et al. 2023) have been employed to produce activated carbons with promising results in MO adsorption. The proliferation of palm waste, propelled by a significant increase in date production within the Arab region from 4.5 million tons in 2000 to 7.12 million tons in 2020, has contributed to an abundance of this agricultural waste (AOAD 2001, 2021). While the literature reveals extensive exploration of various date palm waste components for activated carbon production (Ahmad et al. 2012; Alharbi et al. 2022, Burezq and Davidson 2023), date palm spikelets (DPS), the stalks of the date fruit, remain largely uncharted territory in this domain.

This study pioneers the exploration of DPS as an innovative precursor for developing an activated carbon-derived composite (ZnO@DPS-AC), with a particular focus on enhancing the adsorption efficiency of MO, a prevalent pollutant in wastewater streams. By harnessing the chemical modification potential of zinc chloride followed by a pyrolysis process at 600 °C, this research seeks to unlock the untapped capabilities of ZnO@DPS-AC for MO removal. Employing a suite of characterization tools, including FTIR and XRD, the study delves into the intricate molecular and crystalline structure of ZnO@DPS-AC, establishing a foundational understanding of its adsorptive properties. Additionally, TGA sheds light on the thermal resilience of ZnO@DPS-AC, while SEM reveals its complex surface morphology. Together, these investigative techniques provide a holistic assessment of MO adsorption behavior on ZnO@DPS-AC, spotlighting the transformative potential of repurposing agricultural residues into high-performance adsorbents. This research contributes to the field of environmental engineering by offering a novel, sustainable approach to water treatment and exemplifies the broader applicability of agricultural waste valorization in addressing pressing environmental challenges and circular bioeconomy.

## Materials and methods

### Chemicals

Methyl orange dye (C_14_H_14_N_3_NaO_3_S, Cas: 547–58-0) and a molecular weight of 327.33 g/mol was selected as an adsorbate. Hydrochloric acid (HCl) and sodium chloride (NaCl) were purchased from Sigma-Aldrich. Sodium hydroxide pellets (NaOH) and zinc chloride (ZnCl_2_) were provided by BIOCHEM Chemopharma. All chemicals used were of analytical grade, and deionized water was used throughout the experiments to prepare the reagents.

### Preparation of ZnO@DPS-AC composite

The raw material, date palm spikelets (DPS), was collected from the Biskra region, Algeria. To reduce moisture content and improve handling, the DPS was first dried overnight. Then, it was crushed into fine particles and sieved through an 80-mesh size. Subsequently, the dried PDS was pyrolyzed at 600 °C for 2 h in a one-stage process. The resulting material was washed with 0.1 HCl, dried, and denoted as DPS-Biochar.

The preparation of activated carbon-derived composite from DPS (ZnO@DPS-AC) involves two main steps: impregnation in ZnCl_2_ solution and pyrolysis at high temperature. This procedure adheres to the one reported by (Loke et al. 2022), albeit with minor modifications. The details of the preparation are summarized in Fig. S1. Briefly, about 20 g of sieved PDS was mixed with 20 g of ZnCl_2_ and stirred for 4 h at 65 °C. The mixture was then dried at 105 °C for 24 h. Then, a one-stage pyrolysis process was performed at the same conditions as the DPS pyrolysis. The resulting material was washed with 0.1 M HCl, hot water, and cold water to remove any unreacted materials from the prepared adsorbent. Finally, the washed material was oven-dried at 105 °C for 24 h and denoted as ZnO@DPS-AC composite. Further details concerning the mechanism of ZnO formation are found in the supplementary material.

### Characteristics of ZnO@DPS-AC composite

The physicochemical properties of the ZnO@DPS-AC composite were investigated using various characterization techniques. Thermogravimetric analysis (TGA) with derivative thermogravimetric (DTG) was performed using a thermogravimetry analyzer (TGA–DSC, SDT Q600, TA instruments, USA) to assess the thermal behavior of the raw material. The sample was analyzed under a heating rate of 10 °C/min within a temperature range of 50–800 °C. Scanning electron microscopy (SEM, Thermo Fisher Scientific Prisma E, Czech Republic) equipped with energy-dispersive X-ray spectroscopy (EDS, UltraDry, Thermo Fisher Scientific) was employed to examine the surface morphology and elemental composition of the adsorbents. The average pore diameter was determined from the pore size distribution on the surface of the ZnO@DPS-AC *composite*, utilizing ImageJ software. Fourier transform infrared (FTIR) spectroscopy (FTIR-8400S, Shimadzu Europe) facilitated the identification of the functional groups present in the prepared samples. FTIR spectra were obtained using the KBr disc technique within the range of 400 and 4000 cm^−1^. The structural examination of the ZnO@DPS-AC *composite* involved employing a range of sophisticated techniques. X-ray diffraction (XRD) analysis was carried out utilizing a cutting-edge Bruker D8 Advance diffractometer equipped with CuKa radiation (*λ* = 1.5406 Å) at ambient temperature. The scanning parameters were configured with a range spanning from 10 to 80° and a scan rate of 0.03° s^−1^.

### Determination of point of zero charge (pH_pzc_)

The point of zero charge (pH_PZC_) of the ZnO@DPS-AC composite was determined following the procedure reported by Zhang et al. (2023a). Around 50 mg of ZnO@DPS-AC *composite* was added into each of the 6 volumetric flasks containing 50 mL of 0.1 M of NaCl solution. The initial pH (pHi) of the NaCl solution had been adjusted by 0.1 M HCl or NaOH in the range of 2–12. After stirring in a mechanical shaker for 24 h, the mixture was filtered, and the final pH (pH_f_) was recorded. The pH_PZC_ of ZnO@DPS-AC *composite* is determined by plotting pH_i_–pH_f_ versus pH_i_ of NaCl solutions. The intercept on the *x*-axis is the pH_PZC_.

### Adsorption experiments

The adsorption of methyl orange (MO) onto ZnO@DPS-AC composite was investigated using a series of batch experiments. A stock solution of MO was prepared at a concentration of 500 mg/L. The effects of ZnO@DPS-AC dose (15–100 mg), initial pH of MO solution (2–12), initial MO concentration (50–500 mg/L), contact time (5–360 min), and solution temperature (20–40 °C) were examined. Each experiment involved transferring 50 mL of the MO solution to a 250-mL Erlenmeyer flask and stirring it at 300 rpm. The pH was adjusted using 0.1 M HCl or NaOH solutions. After the adsorption process, the solid–liquid phases were separated through filtration using a 0.45-μm membrane (Whatman filter paper). The remaining MO concentration was determined by UV–vis spectroscopy (Hach DR-600) at its maximum absorbance wavelength of 464 nm. The data obtained from varying the contact time, initial MO concentration, and temperature were subsequently used for kinetic, isotherm, and thermodynamic studies, respectively. All experimental procedures were meticulously replicated to ensure the reproducibility of results. The data presented herein represent the average values with error bars to visualize the associated uncertainty.

### Data calculation and modeling

The efficiency of the adsorption process of MO dye onto ZnO@DPS-AC composite was assessed by calculating the amount of MO dye adsorbed from the solution onto adsorbent at equilibrium *q*_*e*_ (mg/g) and at any time *q*_*t*_ (mg/g) using the Eqs. (1) and (2):

1 $${q}_{e}=\left({C}_{i}-{C}_{e}\right)\frac{V}{m}$$

2 $${q}_{t}=\left({C}_{i}-{C}_{t}\right)\frac{V}{m}$$

where *C*_*i*_, *C*_*e*_, and *C*_*t*_ are the MO concentrations (mg/L) at initial, equilibrium, and time *t*, respectively, *m* is the mass of ZnO@DPS-AC (g), and *V* is the solution volume (L).

Adsorption isotherms and kinetics are crucial as they provide insights into the removal rate of dye and the mechanisms governing the adsorption process. To extract this information, various models were applied to the experimental data. For kinetic analysis, pseudo-first-order (PFO), pseudo-second-order (PSO), Avrami, Elovich, and intra-particle diffusion models were employed. Meanwhile, Langmuir and Freundlich adsorption isotherms were used to investigate the adsorption behavior of MO onto ZnO@DPS-AC. Detailed descriptions of these models are provided in Table S1.

The validity and goodness-of-fit of both isotherm and kinetic models were assessed using statistical parameters. These include the standard deviation (SD), reduced chi-squared (Red-*χ*^2^), coefficient of determination (*R*^2^), and adjusted coefficient of determination (Adj-*R*^2^).

3 $${\text{Red}\_\chi }^{2}= \sum \nolimits_{i=1}^{n}\frac{{\left({q}_{e,\text{exp}}-{q}_{e,\text{cal}}\right)}^{2}}{n-p}$$

4 $$\text{SD}=\sqrt{\left(\frac{1}{n-p}\right)\times \left[\sum \nolimits_{i=1}^{n}{\left({q}_{e,\text{exp}}-{q}_{e,\text{cal}}\right)}^{2}\right]}$$

5 $${R}^{2}=1-\frac{\sum_{i=1}^{n}{\left({q}_{e,\text{exp}}-{q}_{e,\text{cal}}\right)}^{2}}{\sum_{i=1}^{n}{\left({q}_{e,\text{exp}}-{q}_{e,\text{mean}}\right)}^{2}}$$

6 $${R}_{\text{Adj}}^{2}=1-\left(1-{R}^{2}\right). \left(\frac{n-1}{n-p-1}\right)$$

where *q*_*e*,cal_ represents the model’s predicted value for *q*, while *q*_*e*,exp_ signifies the experimentally measured value, *q*_*e*, mean_ refers to the average of all experimental *q* measurements, and *n* and *p* denote the number of experimental data points and model equation parameters, respectively.

The standard Gibbs energy change (Δ*G*°) is a vital parameter calculated using Eq. (7). To gain further insight, the van’t Hoff equation (Eq. (8)) is employed to estimate standard enthalpy change (Δ*H*°) and standard entropy change (Δ*S*°) by plotting lnKe versus (1/*T*(K)). Here, *K*_*e*_ signifies the thermodynamic equilibrium constant, which adheres to the International Union of Pure and Applied Chemistry (IUPAC) recommendation of being dimensionless) (Cohen 2007). Consequently, *K*_*e*_ is evaluated based on the Langmuir constant *K*_L_ (L/mol) using Eq. (9).

7 $$\Delta {G}^{^\circ }=-\text{RTln}{K}_{e}$$

8 $$\text{ln}{K}_{e}=-\frac{\Delta {H}^{^\circ }}{R}\times \frac{1}{T}+\frac{\Delta {S}^{^\circ }}{R}$$

9 $${K}_{e}=\frac{{K}_{\text{L}}\times {C}_{\text{MO}}^{^\circ }}{{\gamma }_{\text{MO}}}$$

where *R* represents the universal gas constant (8.31445 J/(mol. K)), *T* is the solution temperature (K), *C*°_MO_ denotes the standard concentration of MO, and *γ*_MO_ (dimensionless) represents the activity coefficient of MO in solution. Significantly, for dilute solutions, *γ*_MO_ is approximated to 1. Additionally, the IUPAC (Ewing et al. 1995) recommends assuming *C*° as 1 mol/L.

### Reusability and regeneration studies

The regeneration and reuse of the laden adsorbents play a crucial role in both the economic and environmental aspects of water treatment processes. This study examined the reusability of ZnO@DPS-AC composite for methyl orange (MO) dye removal through repeated adsorption and regeneration cycles. Experiments were conducted using 100 mg of ZnO@DPS-AC in 100 mL of MO solution (100 and 200 mg/L) at 20 °C and pH 6 for 3 h. The adsorbent was dried between cycles for reuse. When MO removal efficiency decreased below 50%, the laden ZnO@DPS-AC composite was collected and regenerated. This process involved immersing the laden composite in 0.1 M NaOH at 50 °C for 4 h, followed by thorough washing with deionized water and drying at 105 °C for 24 h. The rejuvenated adsorbent was then employed in the new adsorption cycles.

## Results and discussion

### Characterization

#### Physicochemical characteristics of ZnO@DPS-AC composite

The typical properties of the ZnO@DPS-AC composite are summarized in Table S2. The material demonstrated a high ash content of 65.33%, indicating a significant presence of inorganic compounds. The moisture content was relatively low at 2.42%, suggesting good drying efficiency during preparation. The carbonization yield of 42.11% represents a moderate conversion efficiency from the raw material to the final product. The bulk density of 508.61 g/L indicates a relatively dense structure, which could be advantageous for adsorption processes. These properties were determined using standardized procedures, including international standards for ash and moisture content (ASTM 2009, 2011), and established methods for carbonization yield and bulk density (Ahmed and Dhedan 2012). These properties suggest that ZnO@DPS-AC has a robust composition and structural framework, making it a viable candidate for adsorption applications in wastewater treatment.

#### Thermogravimetric analysis of DPS

**Fig. 1:**
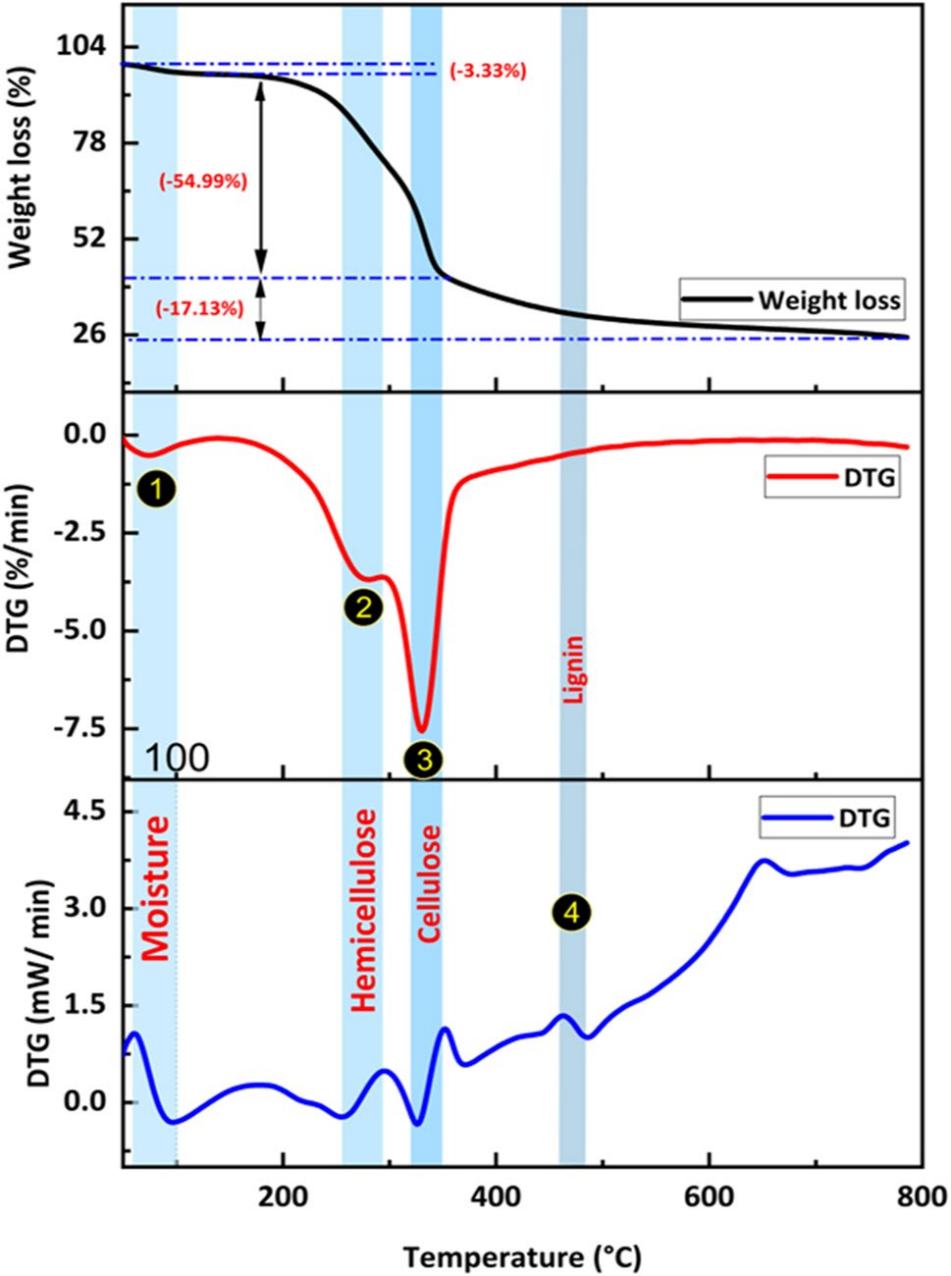
Thermogravimetric analysis, derivative thermogravimetric (DTG), and differential scanning calorimetry (DSC) curves of DPS

Thermogravimetric analysis (TGA) plays a crucial role in designing reactors for the pyrolysis process (Nasser et al. 2016). Therefore, this study employed TGA to assess the thermal stability and decomposition behavior of DPS. A careful analysis of TGA spectra (Fig. 1) revealed a three-stage degradation process for DPS. At the initial stage, a low weight loss occurs between 50 and 113 °C, primarily attributed to the removal of moisture and volatile molecules from the surface. The second stage (183–488 °C) exhibited a significant weight loss corresponding to the decomposition of hemicellulose, cellulose, lignin, and other surface functionalities like lactones. This stage also involves the formation of carboxylic acids and their derivatives, accompanied by releasing CO_2_ as a decomposition product (Bumajdad and Hasila 2023; de Andrade et al. 2015). In the final stage (around 600 °C), the sample exhibits stability, suggesting that the remaining carbon represents the final form of activated carbon (AC). These findings are consistent with previous studies reported in the literature (Kaya and Uzun 2021).

The TGA, derivative thermogravimetric (DTG), and differential scanning calorimetry (DSC) curves are illustrated in Fig. 1. DTG curves exhibit distinct peaks observed at approximately 275 °C, 330 °C, and 465 °C, corresponding to the decomposition of hemicellulose, cellulose, and lignin, respectively. This is in good agreement with established literature values for the individual components (210–325 °C for hemicellulose, 310–400 °C for cellulose, and 160–900 °C for lignin) (Kaya and Uzun 2021). The wider decomposition range observed for lignin is attributed to its complex structure, containing numerous aromatic rings with diverse branches (Tran et al. 2019). Consequently, a temperature of 600 °C was chosen as the pyrolysis temperature for date palm spikelets precursor, as it ensures complete degradation while maximizing char formation.

#### Textural and morphological properties

**Fig. 2:**
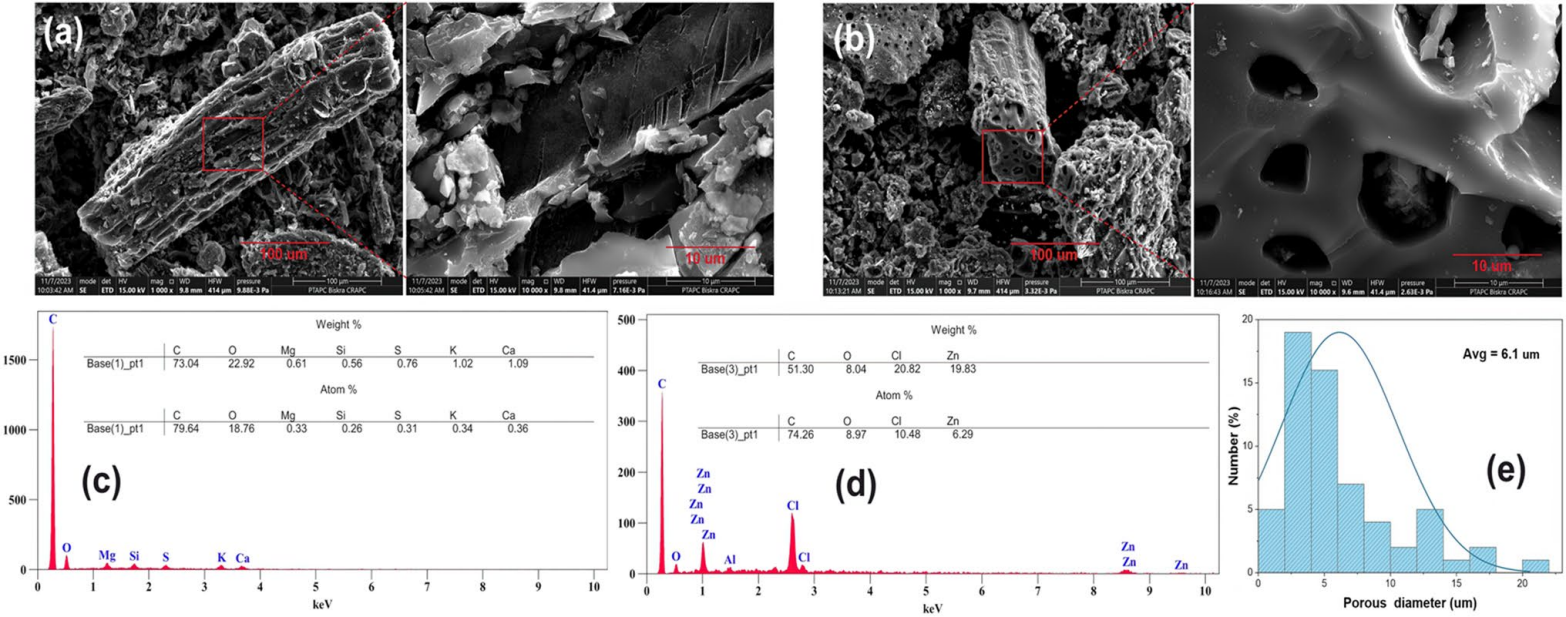
**a**, **c** SEM and EDX of DPS-Biochar sample; **b**, **d** and ZnO@DPS-AC composite; **e** pore size distribution of ZnO@DPS-AC

Scanning electron microscopy (SEM) analysis (Fig. 2) revealed the morphological characteristics and primary components of the DPS-Biochar and ZnO@DPS-AC composite. DPS-Biochar and ZnO@DPS-AC exhibited a heterogeneous structure with a rough and irregular surface. Notably, the biochar surface lacked visible pores (Fig. 2a). At the same time, the ZnO@DPS-AC composite showed pores of varying sizes and shapes (Fig. 2b). This observation is consistent with the previous literature (Zhao et al. 2022; Zubir and Zaini 2020) that reported the crucial role of ZnCl_2_ in significantly increasing the specific surface area and porosity and enhancing adsorption properties of carbon materials. The micrographs further revealed the presence of variously sized grains on the external surface of both samples, consistent with prior research (Uçar et al. 2009). To quantify the porosity of the ZnO@DPS-AC composite, ImageJ software was used to estimate the average pore size based on the SEM image. The analysis yielded an average pore size of approximately 6.1 mm (Fig. 2e).

Energy-dispersive X-ray spectroscopy (EDS) analysis was performed on DPS-Biochar and ZnO@DPS-AC samples to determine their elemental composition. As expected, carbon (C) and oxygen (O) were the dominant elements in both samples, confirming their carbon-rich nature (carbon content > 74%). Notably, the ZnO@DPS-AC composite exhibited detectable amounts of zinc (Zn) (6.29%), indicating the successful formation of Zinc oxide on the surface of the activated carbon. This finding will be further corroborated by XRD analysis. Interestingly, nitrogen (N) was not detected in the samples. However, this absence does not necessarily imply its complete lack. It could be due to either the low initial nitrogen content or the limitations of the EDS detector itself.

#### Crystallographic structures

**Fig. 3:**
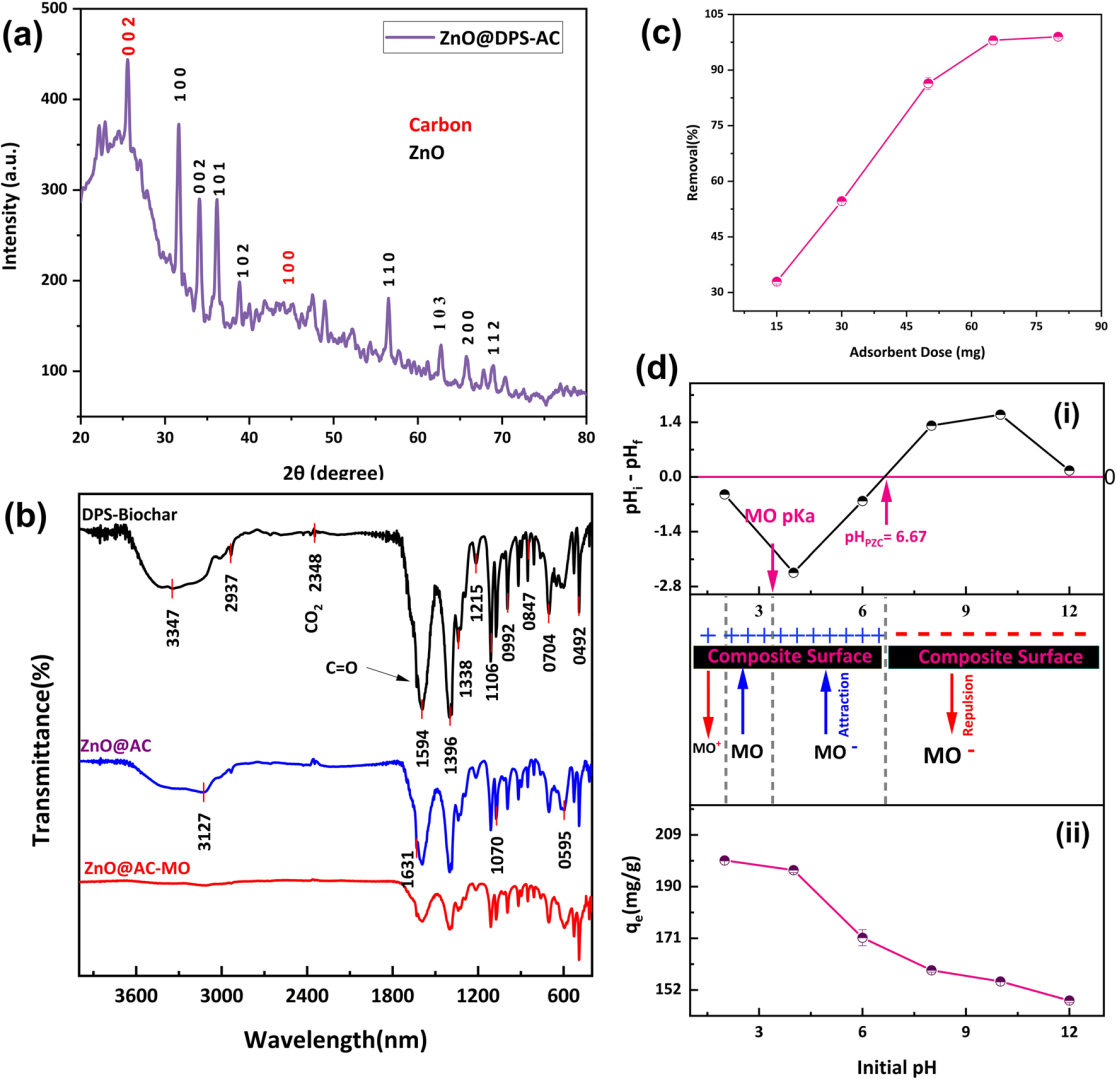
**a** X-ray diffraction pattern of ZnO@DPS-AC composite. **b** FTIR spectra of DPS-Biochar, ZnO@DPS-AC, and the laden ZnO@DPS-AC. **c** The effect of DPS-AC dose on MO adsorption (*C*_0_ = 200 (mg/L), pH = 6, *V* = 50 mL, *T* = 20 °C). **d** (i) Point of zero charge of ZnO@DPS-AC (*C*_NaCl_ = 0.1 M, m/V = 1 (g/L), *T* = 20 °C, *t* = 24 h), (ii) influence of initial pH solution on the removal of MO (*C*_0_ = 200 (mg/L), m/V = 1 (g/L), *T* = 20 °C)

X-ray diffraction (XRD) analysis was employed to gain valuable insights into the crystal structure of the ZnO@DPS-AC composite (Fig. 3a). The results revealed a perfect match between the diffraction pattern of the ZnO@DPS-AC adsorbent and the reference pattern for ZnO with a hexagonal crystal system (JCPDS no. 01–079-0207). This result confirms the presence of ZnO in the composite. Furthermore, the prominent peaks observed at HKI 100, 002, 101, 110, 103, 200, and 112 correspond to the crystal planes of ZnO. Additionally, sharp peaks at approximately 2*θ* = 25.57° and 44.0° were attributed to the (002) and (100) planes of carbon (Naima et al. 2022). These observations suggest the successful formation of a composite material with ZnO embedded within the carbon matrix.

The average crystallite size of ZnO within the ZnO@DPS-AC composite was determined using the Debye–Scherrer equation (Eq. (10)). This equation relates the broadening of X-ray diffraction (XRD) peaks to the size of the crystallite diffracting the X-rays. Employing this approach and considering all prominent ZnO diffraction peaks, the estimated average crystallite size was 37.9 nm.

10 $$D=\frac{k\times \lambda }{\beta \times \text{Cos}(\theta )}$$

where *D* is the average crystallite size (nm), *λ* is the wavelength of the X-ray source (Cu Kα₁ radiation = 0.15406 nm), *k* is the shape factor (Scherrer constant, typically around 0.9), *b* is the full width at half maximum intensity (FWHM) of the diffraction peak in radians, and *q* is the diffraction angle.

#### Surface functional groups

Fourier transform infrared (FTIR) spectroscopy was employed to identify the functional groups present on the surface of DPS-Biochar, ZnO@DPS-AC composite, and the laden ZnO@DPS-AC composite (Fig. 3b). Analysis of the FTIR spectra revealed that the intensities of some peaks for the ZnO@DPS-AC composite were lower compared to DPS-Biochar. This observation can be attributed to the modification process using ZnCl_2_, which acts as a Lewis acid catalyst (Yahya et al. 2015). ZnCl_2_ is known to selectively remove H and O atoms from the precursor, reducing tar formation and increasing carbon content. Elyamny et al. (2022) and Santana et al. (2020) further reported that ZnCl_2_ interacts with organic compounds in the biomass, catalyzing their decomposition and rearrangement. Specifically, Zn^2+^ ions interact with the oxygen in lignin, causing the weakening or breaking of C–O–C, C-O, and CH_2_OH bonds. Additionally, Zn^2+^ coordinates with glycosidic oxygen, facilitating the breakdown of glycosidic linkages in the biomass.

The DPS-Biochar and ZnO@DPS-AC composite revealed the presence of various functional groups on their surfaces, as evidenced by several peaks and bands. The broad band observed in the 3400–3100 cm^−1^ range indicates stretching vibrations of hydroxyl groups likely originating from water, carboxylic acids, phenols, and alcohols (Naima et al. 2022). Aliphatic C-H bonds were identified by the peak at 2936 cm^−1^, suggesting the presence of hydrocarbon chains (Tomul et al. 2021). The overlapping peaks at 1630 cm^−1^ and 1593 cm^−1^ point toward the presence of both carbonyl (C = O) groups in carboxylic acids and C = C bonds in aromatic rings, respectively (Köse et al. 2018). Additionally, a peak around 1393 cm^−1^ signifies the presence of carboxylate groups, further confirmed by the peak at 1331 cm^−1^ attributed to C = O vibrations within these groups (Naima et al. 2022). Peaks within the 1250–1050 cm^−1^ range suggest the presence of functional groups like C-O and C–O–C bonds, potentially associated with carboxylic acids, phenols, alcohols, esters, or ethers (Djezzar et al. 2024; Ninh et al. 2023). Finally, absorption peaks below 1000 cm^−1^ might be attributed to various functionalities, including C-H or C–C vibrations in aromatic rings and alkyl halides (Tavana et al. 2020; Tomul et al. 2021). Notably, no new peaks were observed in the FTIR spectra after DPS modification. This observation could be attributed to the potential overlap of the ZnO stretching vibration with existing peaks in the region below 700 cm^−1^. Prior studies have reported ZnO stretching vibrations at these lower wavelengths (Ahlawat et al. 2023; Alahabadi et al. 2023; Albo Hay Allah and Alshamsi 2023).

### Performance of MO adsorption on ZnO@DPS-AC composite

#### Influence of ZnO@DPS-AC dose

Optimizing the adsorbent dose is paramount for balancing high contaminant removal, efficacy, and cost process (Serban et al. 2023). In this regard, the effect of ZnO@DPS-AC composite dosage on MO removal efficiency was investigated. The experiment employed varying doses of ZnO@DPS-AC composite ranging from 15 to 100 mg (Fig. 3c). The results demonstrate a significant increase in MO removal percentage as the ZnO@DPS-AC dose increased from 15 to 65 mg, with removal efficiency rising from 33 to 98%. This trend can be attributed to the enhanced availability of active sites for MO/ZnO@DPS-AC interaction with each increment in dose. Consequently, the expanding specific surface area facilitates more significant MO removal. Based on these findings, 50 mg of ZnO@DPS-AC composite was selected for further experiments.

#### Influence of initial pH

The initial pH of a solution is a critical factor affecting the adsorption process (Djezzar et al. 2024; Ramutshatsha-Makhwedzha et al. 2022). This influence stems from its impact on several key aspects: the interactions between the adsorbent and the adsorbate molecule, the ionization state of functional groups on the adsorbent’s surface, and the competition between H^+^ and HO⁻ ions for adsorption sites. Another crucial factor is the ionization state of the adsorbate molecule itself. For example, methyl orange (MO, pKa value of 3.46) is a dye whose ionization state depends on the pH. Depending on the solution’s pH, MO can exist in three forms: anionic (MO⁻, pH > 3.46) (Wei et al. 2023), neutral (MO, 2 < pH < 3.4) (Aichour et al. 2022), and cationic (MOH⁺, pH < 2) (Cheng et al. 2023; Loc et al. 2022). Similarly, the surface charge of the adsorbent plays a significant role. The point of zero charge (pH_pzc_) is the pH at which the adsorbent has a net neutral charge. For instance, the ZnO@DPS-AC composite used in this study has a pHpzc of 6.67 (as depicted in Fig. 3d(i)). That means it carries a positive surface charge below this pH and a negative one above.

The synergistic relationship between MO’s ionization state and the adsorbent’s surface charge significantly impacts their interaction. To illustrate this concept, the influence of initial pH on the adsorption of MO onto the ZnO@DPS-AC composite was investigated (Fig. 3d(ii)). The results showed that the maximum adsorption capacity (199.6 mg/L, 99.8% removal) was observed at pH 2. This efficiency gradually decreased to 148.135 mg/L (74.1% removal) at pH 12. These observations can be explained by considering the electrostatic interactions between MO and ZnO@DPS-AC composite at different pH values. Below pH 2, both MO and ZnO@DPS-AC composite are positively charged, leading to minimal interaction due to electrostatic repulsion. In the pH range of 2 to the pKa of MO (3.46), neutral MO molecules interact favorably with the positively charged ZnO@DPS-AC via electrostatic attraction and hydrogen bonding, leading to maximum adsorption. Similarly, between the pKa of MO and the pH_pzc_ of ZnO@DPS-AC (6.67), the anionic MO interacts favorably with the positively charged ZnO@DPS-AC. However, above the pH_pzc_, both MO and ZnO@DPS-AC are negatively charged, resulting in decreased adsorption due to electrostatic repulsion and competition with HO⁻ ions for active sites.

In essence, the optimal adsorption of MO onto ZnO@DPS-AC occurs in highly acidic media (pH = 2). However, ZnO@DPS-AC can still effectively remove MO across a broad pH range (from 2 to 6.7) and demonstrate commendable removal efficiency even in an alkaline environment (pH = 12). This is likely due to other mechanisms and adsorption sites involved in the MO removal process. While a highly acidic environment (pH 2) resulted in the greatest MO adsorption, practical considerations necessitate a more environmentally friendly approach. Discharging large amounts of acidic water after treatment can be harmful, and employing chemicals for large-scale pH adjustments is not recommended. Therefore, considering this balance between efficiency and environmental impact, a pH of 6 was chosen for further experiments. This compromise balances achieving a high removal rate with maintaining environmental sustainability. It is worth mentioning that this choice is consistent with prior studies in which adsorption experiments were carried out at a neutral pH of 7 (Martínez et al. 2023).

#### Influence of ionic strength

**Fig. 4:**
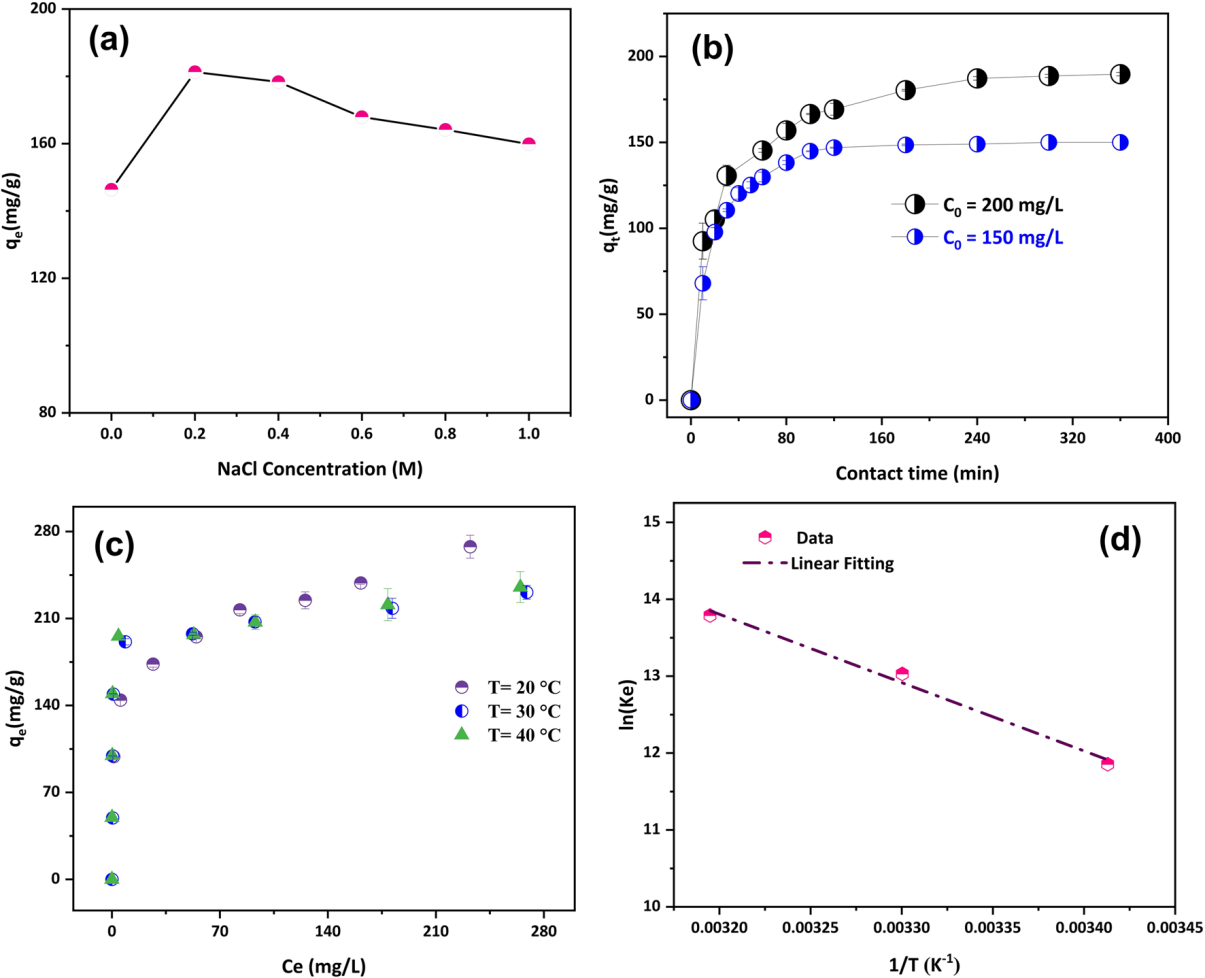
**a** Effect of ionic strength on MO adsorption (*C*_0_ = 200 (mg/L), pH = 6, *m*/*V* = 1 (g/L), *T* = 20 °C); **b** effect of contact time on the adsorption of MO onto ZnO@DPS-AC composite (pH = 6, *m*/*V* = 1 (g/L), *T* = 20 °C); **c** adsorption isotherms of MO onto ZnO@DPS-AC (pH = 6, time 180 (min), *m*/*V* = 1 (g/L)); **d** van’t Hoff plot of MO adsorption onto ZnO@DPS-AC

Understanding the effect of ionic strength on MO adsorption is crucial for two reasons. First, it elucidates the role of electrostatic interactions in the adsorption process. Second, dye-containing wastewater often contains significant salt content, necessitating the investigation of electrolyte effects on MO removal. As reported elsewhere, salt presence can alter dye adsorption by porous carbon, particularly in organic dye wastewater with high salt concentrations (Wang et al. 2021). Therefore, the adsorption capacity of ZnO@DPS-AC composite for MO removal was evaluated across a range of NaCl concentrations (0.2–1 M). As shown in Fig. 4a, the adsorption capacity initially increased from 146.35 to 181.3 mg/L (a 17.5% increase in removal efficiency) with increasing NaCl concentration before experiencing a slight decline. Even at the highest NaCl concentration (1 M), MO adsorption capacity remained above the initial value observed without NaCl. This suggests the effectiveness of ZnO@DPS-AC composite for MO removal under high salt conditions, highlighting the complex impact of NaCl solution on MO adsorption by ZnO@DPS-AC composite.

This observed that increase in adsorption capacity at low-to-moderate NaCl concentrations can be attributed to the synergistic of two opposing effects caused by electrolytes, as suggested by Liu et al. (2009) and Tran et al. (2017a). Electrolytes have two opposite effects on dye removal by carbon material. Firstly, NaCl can neutralize the surface charge of ZnO@DPS-AC, hindering MO adsorption at higher ionic strengths. This reduces available binding sites for charged MO molecules (competition effect) and becomes more pronounced with increasing NaCl concentration. Secondly, NaCl can suppress the dissociation of dye molecules into ionized state, leaving them as neutral species in the solution. These neutral molecules can more readily interact with the ZnO@DPS-AC surface through non-ionic interactions, leading to increased adsorption capacity.

#### Influence of contact time and modeling of adsorption kinetics

Optimizing the contact time between adsorbent and adsorbate presents a paramount operational parameter. This parameter is significant for comprehending adsorption mechanisms and accurately modeling kinetic data. Therefore, the effect of contact time on MO dye adsorption using ZnO@DPS-AC composite was studied in the range from 10 to 360 min. Figure 4b depicts the effect of contact time on the removal efficiency of MO dye at two initial concentrations (200 and 150 mg/L). Initially, a rapid increase in removal efficiency was observed for both concentrations during the first 10 min, exceeding 45%. This corresponds to an adsorption capacity of 92.5 and 68 mg/g for initial dye concentrations of 200 and 150 mg/L, respectively. This high initial adsorption rate is likely attributable to the accessibility of active sites near the adsorbent’s surface. After that, MO uptake gradually increased until reaching equilibrium or saturation between 180 and 240 min for an initial dye concentration of 200 mg/L and 120 min for 150 mg/L. Almost 100% and 94.85% of the initial MO dye (equivalent to 150 and 189.7 mg/g) were removed from the solutions at equilibrium.

Non-linear regression analysis evaluated various kinetic models against the experimental data for MO removal (Fig. S2). The selection criteria prioritized models with the lowest Red-*χ*^2^ value and the highest Adj-*R*^2^ value (closest to 1). Consequently, the fitting order of the models from best to worst fit for the initial dye concentration of 150 mg/L was Avrami > PSO > PFO > Elovich. For the 200 mg/L concentration, the order became Avrami > Elovich > PSO > PFO. Notably, the Avrami model provided the best fit for both initial concentrations, with a half-time of less than 15 min indicating rapid adsorption. Further analysis of the fitting parameters in Table 1 revealed three key trends: (1) a shorter half-time at a lower initial concentration, (2) consistently higher kinetic rate constants for all models at the lower concentration, and (3) dependence of the model’s fitting order on the initial MO concentration. These observations suggest a sensitivity of MO removal kinetics to changes in the experimental conditions, likely due to their influence on the mass transfer driving force. Similar results were found elsewhere in the literature (Chen et al. 2010; Sunthar et al. 2023). The application of the PSO model resulted in a better fit for the experimental data than the PFO model. This finding aligns with several studies in the literature (De Smedt et al. 2024; Loc et al. 2022; Ramath et al. 2023; Tcheka et al. 2024), which suggest that a good fit with the PSO model can be indicative of chemisorption being the rate-limiting step for pollutant removal. Conversely, a better fit with the PFO model is often associated with physisorption as the rate-limiting step. However, some scholars argue against solely relying on kinetic data or fitting for identifying adsorption mechanisms (Kumar 2006; Lima et al. 2016; Tran et al. 2017b). They advocate for incorporating analytical techniques and experimental data of adsorption for a more robust identification.

**Table 1:** Parameters of adsorption kinetics of MO onto ZnO@DPS-AC

Model	Unit	[MO]_0_ = 150 mg/L		[MO]_0_ = 200 mg/L	
		Value	SD	Value	SD
Experimental					
*q*_*e*_	mg/g	150	-	189.7	-
PFO					
*q*_*e*_	mg/g	145.39	2.20	175.68	5.32
*k*_1_	L/min	0.05	0.004	0.048	0.01
*t*_0.5_	min	13.88	-	14.37	-
*R*^2^	-	0.982	-	0.942	-
Adj-*R*^2^	-	0.981	-	0.937	-
Red-*χ*^2^	-	34.41	-	193.56	-
PSO					
*q*_*e*_	mg/g	158.48	1.14	193.19	3.74
*k*_2_	g/mg.min	4.96 × 10^−4^	2.31 × 10^−5^	3.52 × 10^−4^	4.34 × 10^−5^
*t*_0.5_	min	12.72	-	14.69	-
*R*^2^	-	0.998	-	0.9863	-
Adj-*R*^2^	-	0.997	-	0.985	-
Red-*χ*^2^	-	4.67	-	45.98	-
Avrami					
*q*_*e*_	mg/g	150.33	0.87	200.93	6.11
*k*_AV_	min^−1^	0.0498	0.001	0.032	0.004
*n*_AV_		0.68	0.02	0.46	0.04
*t*_0.5_	min	11.71	-	14.19	-
*R*^2^	-	0.9988	-	0.997	-
Adj-*R*^2^	-	0.9986	-	0.996	-
Red-*χ*^2^	-	2.51	-	11.62	-
Elovich					
*α*	mg/(g.min)	111.23	62.57	70.93	16.1
*β*	g/mg	0.046	0.01	0.034	0.002
*R*^2^	-	0.961	-	0.992	-
Adj-*R*^2^	-	0.958	-	0.992	-
Red-*χ*^2^	-	74.18	-	25.24	-
Intra-particle diffusion					
*Stage I*					
*k*_IPD_	mg/ [g. min^0.5^]	20.68	1.02	24.57	4.43
*C*	mg/g	1.29	3.97	3.35	14.02
*R*^2^	-	0.995	-	0.969	-
Adj-*R*^2^	-	0.993	-	0.937	-
SD	-	35.67	-	207.68	-
*Stage II*					
*k*_IPD_	mg/ [g. min^0.5^]	6.74	0.07	6.48	0.57
*C*	mg/g	77.64	0.6	97.09	5.52
*R*^2^	-	0.9996	-	0.970	-
Adj-*R*^2^	-	0.9995	-	0.963	-
SD	-	0.14	-	47.83	-
*Stage III*					
*k*_IPD_	mg/ [g. min^0.5^]	0.39	0.06	0.71	0.04
*C*	mg/g	142.91	0.91	176.37	0.68
*R*^2^	-	0.9375	-	0.9969	-
Adj-*R*^2^	-	0.9167	-	0.9938	-
SD	-	0.41	-	0.01	-

To gain a deeper understanding of the mechanisms governing the adsorption of MO dye onto ZnO@DPS-AC composite, this study employed the Weber-Morris intra-particle diffusion model. PSO, PFO, and other models often fail to provide insights into the rate-limiting steps and reaction pathways that govern adsorption (Tran et al. 2017b). The Weber-Morris model offers a more nuanced perspective, revealing a multistep adsorption mechanism involving film diffusion, intra-particle diffusion, and attachment/equilibrium (Fig. S3). The initial stage involves film diffusion, where MO molecules migrate from the bulk solution to the external surface of the ZnO@DPS-AC particles. Subsequently, MO molecules diffuse into the pores via intra-particle diffusion. Finally, the MO molecules bind to active sites within the pores, reaching an equilibrium state. The model’s rate constant values (kIPD) follow the sequence *k*_IPDI_ > *k*_IPDII_ > *k*_IPDIII_, indicating a higher initial adsorption rate that slows down over time. The intercept parameter (*C*) values are also consistently higher for an initial MO concentration of 200 mg/L compared to 150 mg/L. This parameter reflects the thickness of the boundary layer surrounding the ZnO@DPS-AC particles. A thicker boundary layer (higher *C*) can hinder external mass transfer from the solution but promote internal mass transfer within the adsorbent (Ahmad et al. 2021).

#### Influence of initial concentration and isotherm behaviors

The effect of the initial dye concentration on the adsorption process of MO was investigated, as depicted in Fig. 4d. The results show that the increase in uptake of MO increases as the initial concentration increases. This can be attributed to the concentration gradient, which is a driving force in overcoming the mass transfer barrier between ZnO@DPS-AC composite and the MO solution. At low initial concentrations (50–150 mg/L), the adsorbent effectively removed the MO, with a removal percentage of 96.2–99%. This observation indicates a strong adsorbent-MO interaction, with abundant unoccupied active sites available for adsorption. As the initial MO concentration increased (200–500 mg/L), a gradual increase in the quantity adsorbed was noticed. This is likely because fewer sites were available at higher concentrations. A similar adsorption trend has been reported in the literature elsewhere (Tcheka et al. 2024). Tcheka et al. (2024) utilized *Hyphaene thebaica* fruit shells to prepare soda-activated *Hyphaene thebaica* (HTBS) and *Hyphaene thebaica*-derived biochar (HTBC) for the removal of MO dye. The isotherm analysis was performed at 45 °C with an adsorbent mass of 30 mg and pH 2 and with initial dye concentrations ranging from 25 to 300 mg/L. The results revealed an L-type adsorption isotherm, indicating a strict plateau in adsorbed MO for both materials after exceeding 200 mg/L. They suggested that the adsorbent surfaces are capable of effectively adsorbing dye molecules only when the initial concentrations do not exceed 200 mg/L.

The equilibrium adsorption of MO dye onto ZnO@DPS-AC composite was investigated using various initial dye concentrations (50–500 mg/L) at three different temperatures (20 °C, 30 °C, and 40 °C) and a constant solid/liquid ratio (1 g/L). Adsorption isotherms, which depict an adsorbent’s performance at equilibrium, were employed to analyze the data (Fig. S2). The isotherm shapes were classified as H-type, indicating strong adsorption affinity between ZnO@DPS-AC and MO (Giles et al. 1974). To further assess the adsorption process, Langmuir and Freundlich isotherm models were applied to fit the experimental data (Fig. S2). The parameters were calculated using a non-linear regression (Table 2). The Freundlich isotherm provided a better fit at 20 °C, whereas the Langmuir isotherm was more suitable at 30 °C and 40 °C. The inherent assumptions of each model can explain this observation. The Langmuir isotherm assumes monolayer adsorption on a homogeneous surface, while the Freundlich isotherm describes multilayer adsorption on a heterogeneous surface with possible adsorbate interactions (Al-Ghouti and Da’ana 2020). Therefore, the results suggest that MO dye adsorption primarily occurs via a heterogeneous and multilayer mechanism at lower temperatures (20 °C). As the temperature increases (30 °C and 40 °C), the adsorption process might favor a more homogeneous surface, with the possibility of a monolayer mechanism.

**Table 2:** Adsorption isotherms parameters for MO removal onto ZnO@DPS-AC composite

		*T* = 20 °C		*T* = 30 °C		*T* = 40 °C	
Model	Unit	Value	SD	Value	SD	Value	SD
Langmuir							
*q*_*m*_	mg/g	226.81	10.98	214.77	9.97	215.29	5.42
*K*_L_	L/mg	0.43	0.15	1.39	0.35	2.98	0.46
*R*^2^	-	0.932	-	0.942	-	0.983	-
Adj-*R*^2^	-	0.923	-	0.934	-	0.980	-
Red-*χ*^2^	-	582.5	-	441.26	-	134.79	-
Freundlich							
*K*_F_	(mg/g)/(mg/L)^n^	87.93	6.829	116.38	14.77	126.84	13.47
1/*n*	-	0.201	0.02	0.128	0.029	0.11	0.024
*R*^2^	-	0.981	-	0.887	-	0.899	-
Adj *R*^2^	-	0.978	-	0.871	-	0.885	-
Red-*χ*^2^		165.1	-	863.44	-	791.21	-

The Langmuir *q*_*m*_ values, representing the maximum adsorption capacity, were 226.81 mg/g (20 °C), 214.77 mg/g (30 °C), and 215.29 mg/g (40 °C). The Freundlich constant (1/*n*) values between 0 and 1 also indicate favorable adsorption (Tran et al. 2017b). The high *Q*_max_ value further emphasizes the potential of ZnO@DPS-AC composite as an effective dye removal agent, as will be compared to *q*_*m*_ values of various adsorbents reported in the existing literature (Table 3).

**Table 3:** Maximum monolayer adsorption capacity (*q*_*m*_) of MO at different adsorbents

Adsorbent	Adsorption conditions				*Q*_0(max)_ (mg/g)	Ref
	*C*_0_ (mg/L)	*m*/*V* (g/L)	*T* (°C)	pH		
*Hyphaene thebaica* biochar (HTBC)	25–300	0.6	45	2	195.15	(Tcheka et al. 2024)
Commercial activated carbon (CAC)	5–100	0.1	25	3	129.3	(Serban et al. 2023)
Activated pine cone (APC) biochar	100–400	0.8	25	2	80.37	(Kaya and Uzun 2021)
Wood sawdust-derived composite (ZnO@AC)	10–200	1.2	20	3	44.5	(Sayed et al. 2024)
CAC-derived composite (ZnO-NP@AC) Orange Peel-derived composite (ZnO-NP@AC)	20–100	0.4	27	4	138.8 196.1	(Ahlawat et al. 2023)
SiO_2_ nano-particles	5–250	1.6	-	4	69.40	(Zhang et al. 2023b)
Date palm spikelets-derived composite (ZnO@DPS-AC)	50–500	1	20	6	226.81	This work

#### Thermodynamics study

The influence of temperature on the adsorption of MO onto ZnO@DPS-AC composites was investigated to elucidate the underlying thermodynamic parameters governing the adsorption process. As mentioned previously, thermodynamic parameters were calculated using the equations described in the “Data calculation and modeling” section, and the results are summarized in Table 4. A plot based on the van’t Hoff equation can be found in Fig. 4d of the supplementary material. According to Table 4, the increasing *K*_L_ constant with temperature suggests an endothermic process. This is further confirmed by the positive value of Δ*H*° (> 0 kJ/mol). Furthermore, the negative values of Δ*G*° indicate a thermodynamically favorable adsorption process, even though the positive Δ*S*° signifies increased randomness at the solid-solution interface during adsorption. The magnitude of Δ*H*° can provide insight into the nature of the adsorption mechanism. According to Chang and Thoman Jr. (2014) and Zhang et al. (2023c), the magnitude of Δ*H*° in physical adsorption (Δ*H*° < 31 kJ/mol) is less than that observed in chemical adsorption (Δ*H*° ≥ 80 kJ/mol). In this study, the Δ*H*° value was 73.911 kJ/mol. Considering both the Δ*H*° value and the isotherm studies performed at different temperatures in the “Influence of initial concentration and isotherm behaviors” section (Freundlich isotherm at 20 °C and Langmuir isotherms at 30 °C and 40 °C), the removal of MO dye by the ZnO@DPS-AC composite likely transpires via a combined mechanism. This mechanism encompasses physical and chemical adsorption processes within the investigated temperature range.

**Table 4:** Values of the thermodynamic parameters for MO adsorption onto ZnO@DPS-AC

*T* (K)	*K*_L_ (L/mg)	*K*_*e*_	Δ*G*° (kJ/mol)	Δ*H*° (kJ/mol)	Δ*S*° (J/(mol.K))	van’t Hoff equation
293	0.43	140,817.37	− 28.88	73.9	351.3	*Y* = − 8889.5 *X* + 42.3 *R*^2^ = 0.989
303	1.39	456,183.46	− 32.83			
313	2.98	974,788.74	− 35.89			

### Application of ZnO@DPS-AC composite for wastewater treatment and other pollutants

This study investigated the application of ZnO@DPS-AC composite for treating industrial wastewater and its effectiveness in improving various water quality parameters. Industrial wastewater from diverse industrial and commercial processes poses a significant environmental challenge. This wastewater comprises water utilized during production, often laden with contaminants such as organic substances, heavy metals, and hazardous chemicals. To address this issue, the efficacy of ZnO@DPS-AC composite was examined. The wastewater was collected from a local cable production facility (ENICAB, Biskra). The experimental procedure involved agitating 500 mL of industrial wastewater with 500 mg of ZnO@DPS-AC composite adsorbent for 120 min under ambient conditions.

The results showed notable improvement in various water quality parameters following treatment with the ZnO@DPS-AC composite. This improvement was evident in the conductivity reduction from 1272 to 872 µS/cm, suggesting a decrease in dissolved ions. Similarly, total alkalinity (TAC) and salinity (Sal) decreased from 400 mg/L and 0.6% to 200 mg/L and 0.3%, respectively, indicating the removal of carbonate and bicarbonate ions. A decrease in total dissolved solids (TDS) from 560 to 430 mg/L further supported the removal of dissolved salts and organic matter. Meanwhile, the biological oxygen demand (BOD) decreased (1400 to 840 mg/L), suggesting the partial removal of biodegradable organic matter. Suspended solids (SS) were eliminated, and total hardness (TH) displayed a significant decrease, indicating the removal of hardness-causing ions. Additionally, turbidity significantly improved (3.16 NTU to 0.2 NTU), suggesting enhanced water clarity. Furthermore, the ZnO@DPS-AC composite effectively reduced the concentrations of specific ions, including calcium (Ca^2+^), magnesium (Mg^2+^), nitrate (NO^−^_3_), and ammonium (NH₄^+^). This selective adsorption process contributed to enhanced water quality. Detailed analyses of other physiochemical parameters are provided in Table S3. Overall, the ZnO@DPS-AC composite demonstrates its potential as a cost-effective, environmentally friendly solution for treating industrial wastewater by transforming it into a clean effluent that adheres to discharge regulations.

**Fig. 5:**
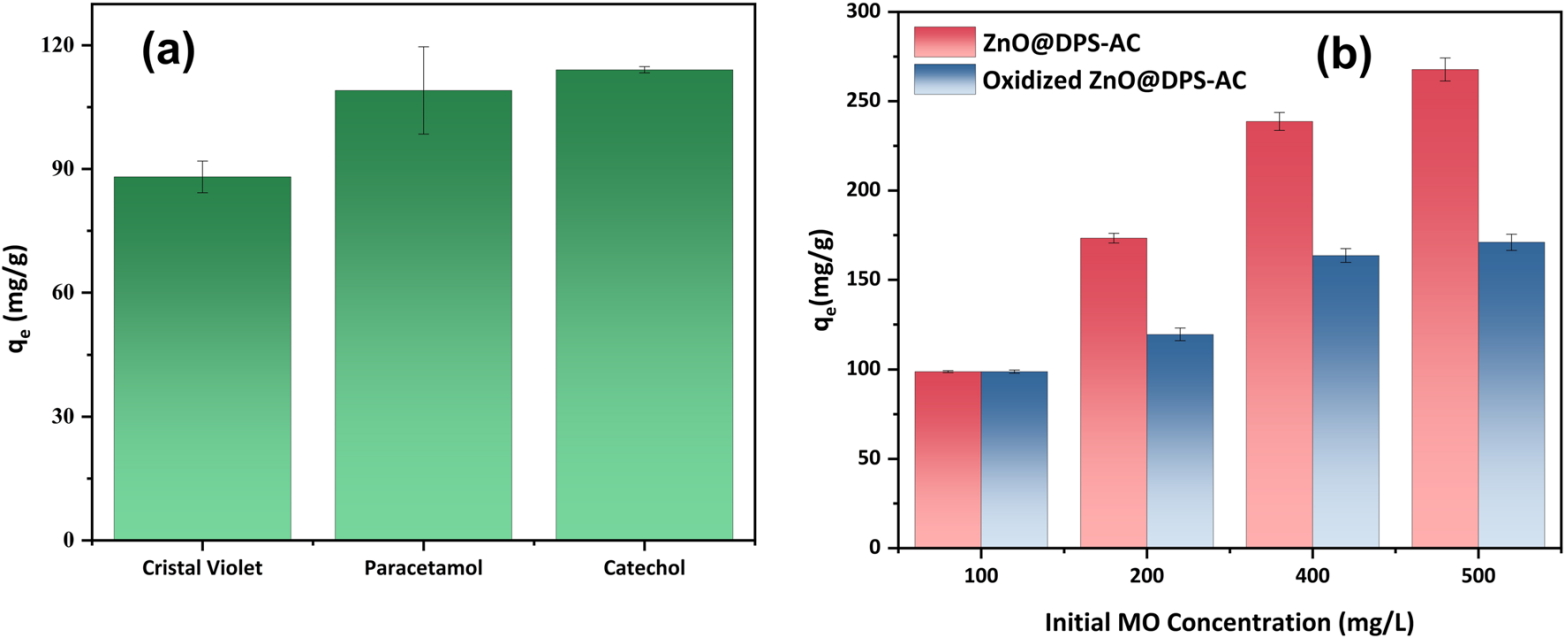
**a** Potential of ZnO@DPS-AC composite toward various kinds of contaminants (*m*/*V* = 0.6 (g/L), *C*_0_ = 100 (mg/L), time = 180 min, *T* = 20 °C, and pH = 7 for crystal violet and 6 for the other contaminants); **b** comparison of adsorption capacity of ZnO@DPS-AC and Oxidized ZnO@DPS-AC for MO removal (*m*/*V* = 1 (g/L), time = 180 min, *T* = 20 °C, and pH = 6)

The ability to remove additional pollutants was investigated to assess the versatility of ZnO@DPS-AC composite in treating a broader range of contaminants commonly found in industrial wastewater. These pollutants included crystal violet, paracetamol, and catechol. As illustrated in Fig. 5a, the ZnO@DPS-AC composite effectively removed all three tested pollutants, demonstrating its potential for application in various wastewater treatment scenarios beyond removing the previously mentioned contaminants. This versatility highlights the promising capabilities of ZnO@DPS-AC composite as a robust adsorbent for industrial wastewater treatment.

### Reusability and regeneration of ZnO@DPS-AC composite

**Fig. 6:**
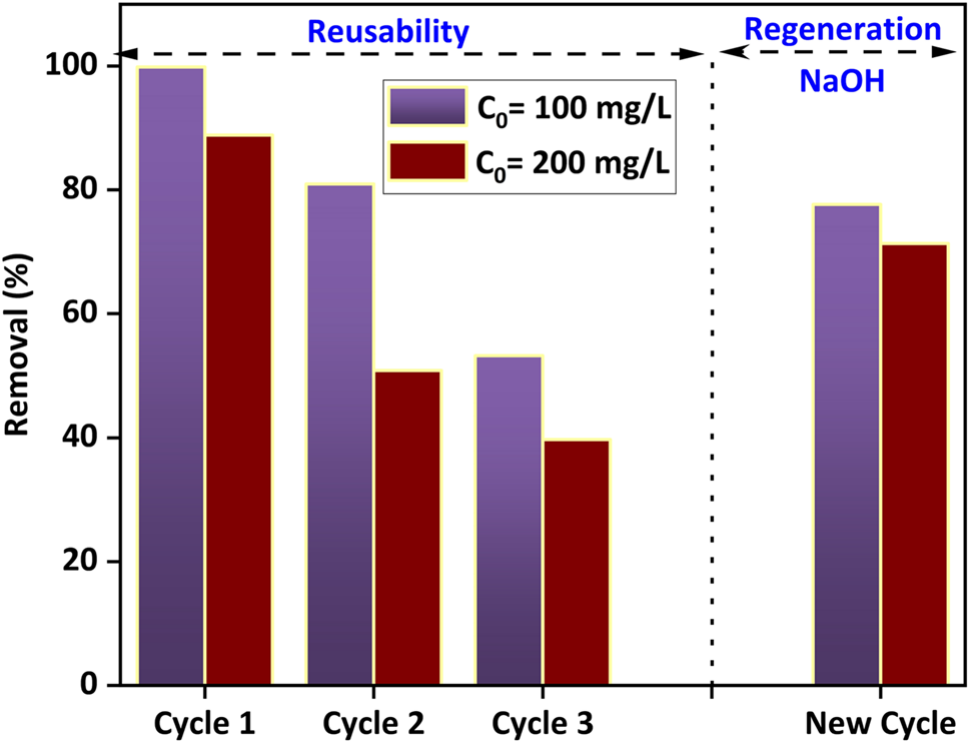
Reusability and regeneration studies for the prepared adsorbent (*m*/*V* = 1 (g/L), time = 180 min, *T* = 20 °C, and pH = 6)

A thorough evaluation of an adsorbent’s reusability is essential to ensure its economic viability in large-scale applications. The reusability and regeneration potential of the ZnO@DPS-AC composite were evaluated and are presented in Fig. 6. The composite exhibited significant adsorption capacity for methyl orange (MO) over three consecutive cycles. However, removal efficiency decreased from 88 to 39% for 200 mg/L initial dye concentration and from 99 to 53% for 100 mg/L. This reduction in performance can be attributed to the gradual depletion of active adsorption sites on the composite surface. Following the third cycle, the laden adsorbent was regenerated using 0.1 M NaOH. Post-regeneration, the composite demonstrated renewed effectiveness, achieving removal efficiencies of approximately 77% and 71% for initial dye concentrations of 200 mg/L and 100 mg/L, respectively. These findings suggest that the ZnO@DPS-AC composite represents a cost-effective and recyclable adsorbent for MO dye removal in water treatment applications.

### Proposed mechanisms of MO adsorption onto ZnO@DPS-AC composite

**Fig. 7:**
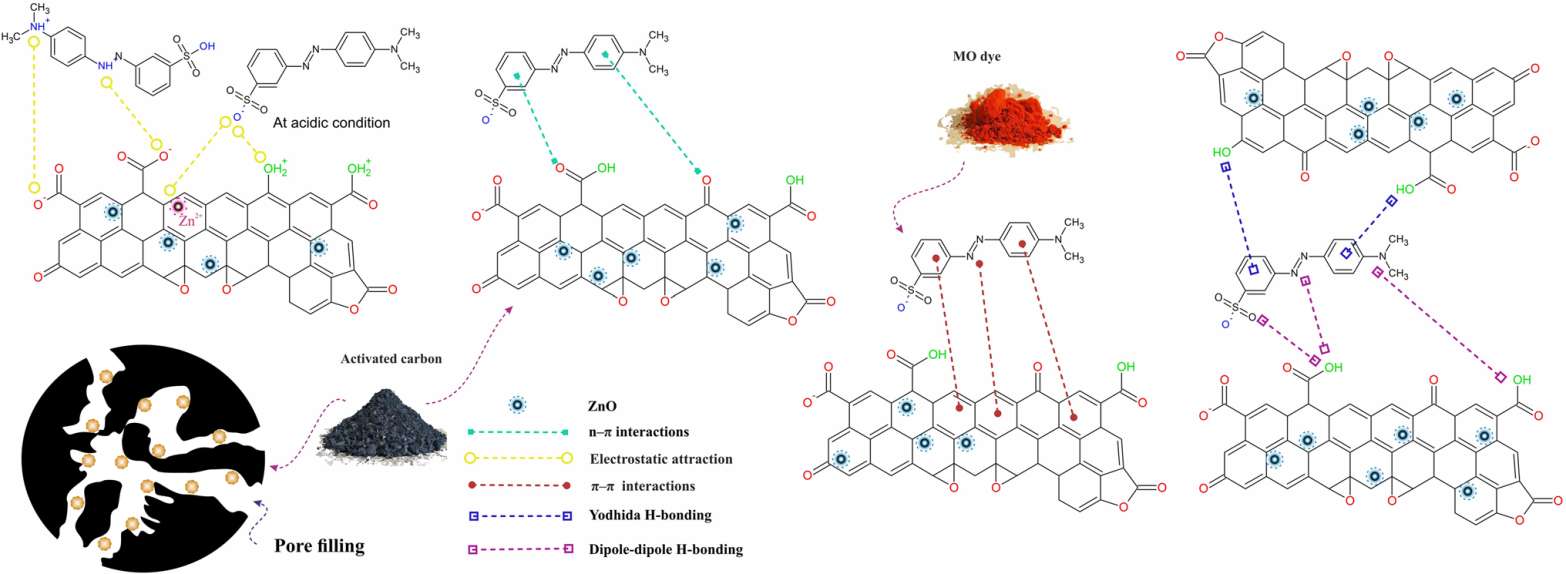
Proposed mechanisms for MO adsorption onto ZnO@DPS-AC

The adsorption of MO dye onto the ZnO@DPS-AC composite is a complex process governed by multiple interactions between the MO molecules and the composite surface, as illustrated in Fig. 7. Electrostatic attraction occurs between MO’s sulfonate group (SO3-) and protonated ZnO@DPS-AC’s surface group. Additionally, impregnated Zn^2+^ ions on the active carbon surface might contribute to MO adsorption through electrostatic attraction (Zbair et al. 2018; Zhang et al. 2016). However, high removal, even at high pH (12) suggests other mechanisms dominate. Furthermore, the fact that MO adsorption capacity remains above the initial value even at the highest NaCl concentration (1 M) further indicates that electrostatic interactions are not the primary mechanism.

FTIR analysis revealed a significant decrease in the intensity of the -OH band after MO adsorption, suggesting hydrogen bonding as a critical mechanism for MO removal by ZnO@DPS-AC. Two types of hydrogen bonding interactions are likely to be involved (Tran et al. 2020). The first is dipole–dipole H-bonding, which occurs between the hydroxyl groups on ZnO@DPS-AC and the MO’s oxygen and nitrogen atoms. The second is Yoshida H-bonding, which involves interaction between the -OH groups on ZnO@DPS-AC and the aromatic rings in MO. Additionally, n-π interactions might contribute to some extent. In n-π interactions, the carbonyl oxygen on the ZnO@DPS-AC surface acts as an electron donor, while the aromatic rings of MO act as electron acceptors (Tran et al. 2020). The weaker decrease in C-O and C = O peak intensities compared to the -OH band after MO adsorption suggests a less significant role for n-π interactions compared to hydrogen bonding.

Another important mechanism is π-π interaction. This attractive force arises between the π-electrons of aromatic rings in ZnO@DPS-AC and those in the aromatic rings or azo groups (-N = N-) of MO (Tcheka et al. 2024). The significant change observed in the C = C peak intensity at 1593 cm^−1^ upon MO adsorption supports the presence of these interactions. H_2_O_2_ oxidation was employed to introduce oxygen functional groups onto the surface of the ZnO@DPS-AC composite to strengthen this evidence further. This procedure reduces the π-electron density of individual graphene layers (Tran et al. 2020), weakening the π-π interactions between ZnO@DPS-AC and MO. As expected, the adsorption capacity of MO onto the oxidized ZnO@DPS-AC composite significantly decreased compared to the original ZnO@DPS-AC composite (Fig. 5b), reinforcing the vital role of π-π interactions in the adsorption mechanism.

The ZnO@DPS-AC composite used in this study exhibits an average pore diameter of 6.1 μm (Fig. 2e). This characteristic facilitates the adsorption of MO molecules through a pore-filling mechanism. Subsequently, efficient diffusion of these MO molecules into the internal pore network of the adsorbent, mainly through the micropores, becomes crucial. Prior research by (Wei et al. 2023) established the optimized dimensions of MO molecules via density functional theory (DFT) to be 1.561 nm in length, 1.023 nm in width, and 0.654 nm in height. These dimensions allow for the free diffusion of MO molecules within the pores of the ZnO@DPS-AC composite. After MO diffusion, adsorption onto the active sites occurs. Therefore, pore filling likely plays a significant role in the MO adsorption mechanism employed by the ZnO@DPS-AC composite for efficient MO removal.

In conclusion, π-π interactions, hydrogen bonding, pore filling, and possibly chemical bonds (as discussed in the “Thermodynamics study” section) are the significant contributors to MO removal by the ZnO@DPS-AC composite. On the other hand, electrostatic attractions and n-π interactions are minor contributors to MO adsorption onto the prepared adsorbent.

### Economic cost analysis

Cost analysis of adsorbents is critical in evaluating their feasibility for commercial use and potential scale-up from pilot to industrial production (Bassam et al. 2024). This assessment determines the economic viability of the activated carbon production process. It is essential to thoroughly examine key factors influencing costs, including raw material availability, treatment conditions, and other process requirements (Yihunu et al. 2019). For the ZnO@DPS-AC composite, the cost analysis encompasses raw materials, transportation, labor, and adsorbent production expenses. The precursor, being an abundant agricultural waste, incurs no cost. Moreover, the laboratory-scale production process is straightforward, requiring minimal complex technologies or labor. Consequently, the precursor, transportation, and labor expenses are considered negligible.

Having established the significance of cost analysis in evaluating adsorbent feasibility, the subsequent analysis focuses on the specific cost components associated with ZnO@DPS-AC composite production. The following breakdown elucidates the various costs involved in the production process, providing a comprehensive overview of the economic factors in adsorbent development. The total production cost of the adsorbent is calculated using the following equation:

10 $$\text{Production cost}= {\text{CG}}_{C}+ {S}_{C} + {P}_{C} + {R}_{C}+ {W}_{C}$$

CG_*c*_ represents the crushing and grinding cost, *S*_*c*_ is the stirring cost, *P*_*c*_ is the pyrolysis cost, and *W*_*c*_ is the washing cost. In the context of laboratory-scale production, certain costs are minimized or eliminated. For instance, raw material crushing is performed manually, and washing costs are negligible due to the availability of deionized water. The primary expenses in this setting are associated with drying, stirring, and pyrolysis, which can be quantified using (Eq. (12)) (Syafiuddin et al. 2019). The overall costs for the preparation of the ZnO@DPS-AC composite are provided in Table 5.

11 $$\text{Activity cost}=\text{Time }\left(\text{h}\right)*\text{ Equipment Power }\left(\text{kW}\right)*\text{ electricity tariff in Algeria}$$

**Table 5:** Cost estimation for preparing 1 kg of ZnO@DPS-AC composite

Details	Cost (USD/Kg)
Grinding	0.0059
Stirring	0.0369
Pyrolysis	0.1578
Drying	0.3791
ZnCl_2_ (LG)	80–105*
ZnCl_2_ (IG)	0.67–0.95**
Total	80.58–100.58 (LG)
	1.25–1.56 (IG)
Cost of removing 1g of MO	0.355–0.465 (LG)
	0.0055–0.00687 (IG)

^*^LG represents the price of ZnCl_2_ laboratory grade according to the Algerian market; **IG is the price of ZnCl_2_ industrial grade according to the trading made in China website

The cost breakdown analysis reveals that ZnCl₂ is the primary expense, while other costs are relatively minor. Using laboratory-grade materials, the estimated total cost for producing 1 kg of ZnO@DPS-AC composite ranges from 80.58 to 100.58 USD/kg. However, this cost significantly decreases to 1.25–1.56 USD/kg when using ZnCl₂ of industrial-grade. The higher laboratory cost is attributed to the high-purity ZnCl₂, whereas industrial applications typically employ chemicals of comparable quality but lower cost to reduce production expenses. In industrial-scale production, additional factors must be considered beyond basic production expenses. These include the precursor, transportation, labor, taxes, maintenance, administration, utilities, industrial gases, and waste management (Salem et al. 2023). The increased production volume at this scale leads to reduced operating costs and expenses, making the total production cost of ZnO@DPS-AC composite sustainable at 1.25–1.56 USD/kg. This cost is lower than that of activated carbon from coal, approximately 1.7 USD/kg.

Having established the production costs of ZnO@DPS-AC composite, evaluating its economic efficiency in terms of pollutant removal capacity is essential. This assessment provides a more comprehensive picture of the adsorbent’s cost-effectiveness compared to alternative materials. To contextualize the economic viability of ZnO@DPS-AC composite, its adsorption capacity must be considered concerning its production cost. With an adsorption capacity of 226.81 mg/g (Table 3), the expense for removing 1 g of methyl orange dye using this composite ranges from 0.0055 to 0.00687 USD/g. This cost-efficiency is particularly notable when compared to other adsorbents in the field. For instance, geopolymer powder (GPP) and sodium alginate beads (Alg/GPP), developed from red clay waste for methyl orange removal, have reported costs of 5.948 and 2.981 USD/kg, respectively (Bassam et al. 2024). Despite their comparable adsorption capacities of 94.78 and 224.85 mg/g, the removal cost for 1 g of methyl orange dye with these materials is significantly higher at 0.0627 and 0.0133 USD/g, respectively. These comparisons underscore the superior cost-effectiveness of the ZnO@DPS-AC composite in the context of methyl orange dye removal.

### Environmental friendliness analysis

The ZnO@DPS-AC composite demonstrates significant environmental advantages and is consistent with circular bioeconomy principles and sustainable waste management. Using abundant agricultural waste (DPS) as the primary raw material exemplifies resource efficiency and waste valorization. The one-stage pyrolysis process at 600 °C and minimal chemical usage in preparation contribute to a potentially reduced carbon footprint compared to conventional activated carbon production methods.

With a superior maximum monolayer adsorption capacity of 226.811 mg/g for methyl orange under mild conditions (pH 6), the ZnO@DPS-AC composite outperforms many recent adsorbents (Table 3). This high efficiency translates to more effective wastewater treatment with lower adsorbent usage. The composite’s reusability over multiple adsorption cycles and effective regeneration using 0.1 M NaOH further enhance its environmental profile by extending its lifecycle and reducing waste generation.

Economically, the estimated production cost of 1.25–1.56 USD/kg (using industrial-grade ZnCl₂) is competitive with conventional activated carbons. The cost-effectiveness of removing 1g of methyl orange (0.0055–0.00687 USD/g) surpasses that of other reported adsorbents, potentially leading to more widespread adoption. Using available waste enables localized production, potentially reducing transportation-related emissions.

In conclusion, the ZnO@DPS-AC composite represents a significant advancement in environmentally friendly wastewater treatment, embodying principles of waste valorization, energy efficiency, and sustainable materials design. Its superior performance under mild conditions, coupled with economic viability, underscores its potential for efficient and environmentally benign water treatment solutions. Future life cycle assessments could further quantify these environmental benefits, providing a more comprehensive understanding of the material’s sustainability profile compared to other adsorbents and treatment technologies.

## Conclusion

This study demonstrates the successful synthesis of ZnO@DPS-AC composite from date palm spikelets, offering a sustainable solution for water treatment within the circular bioeconomy framework. The composite exhibited high carbon content (> 74%), diverse functional groups, and extensive porosity, enabling rapid MO removal (45% within 10 min, up to 99% under optimal conditions). With a maximum adsorption capacity of 226.811 mg/g, ZnO@DPS-AC outperforms many existing adsorbents. Its versatility was evident in effectively removing various pollutants beyond MO, including crystal violet, paracetamol, and catechol, and its promising performance in real wastewater treatment scenarios. The low-cost production process further enhances its potential for large-scale applications. Future research should focus on optimizing the production process for industrial-scale implementation, investigating the composite’s effectiveness against a broader range of pollutants, exploring modifications to enhance selectivity and regeneration capabilities, and conducting long-term studies on the adsorbent’s performance and stability. In conclusion, this study not only introduces an innovative, cost-effective adsorbent but also exemplifies the potential of agricultural waste valorization in addressing pressing environmental challenges. By bridging the gap between waste management and water treatment, ZnO@DPS-AC represents a significant step toward a more sustainable and circular approach to resource utilization and environmental protection.

### Supplementary Information

Below is the link to the electronic supplementary material.

Supplementary file1 (DOCX 9027 KB)

## Data Availability

Data will be made available on request. The data discussed in the manuscript are included in the text itself.
