# Supplementary material for: Valorizing date palm spikelets into activated carbon-derived composite for methyl orange adsorption: advancing circular bioeconomy in wastewater treatment—a comprehensive study on its equilibrium, kinetics, thermodynamics, and mechanisms
Source: Environ Sci Pollut Res Int. 2024 Aug 3;31(38):50493–512. doi: 10.1007/s11356-024-34581-3 (PMC11364697; doi:10.1007/s11356-024-34581-3)
Supplement: Supplementary file 1 — Supplementary file1 (DOCX 9027 KB) [file 11356_2024_34581_MOESM1_ESM.docx]

**Supplementary Data**

**Valorizing** **Date Palm Spikelets** **into Activated Carbon-derived Composite for Methyl Orange Adsorption: Advancing Circular Bioeconomy in Wastewater Treatment – A Comprehensive Study on Its Equilibrium, Kinetics, Thermodynamics, and Mechanisms**

Mazen S.F. Al-Hazeef ^1^, Amel Aidi ^1,2^, Lynda Hecini ^3,4^, Ahmed I. Osman ^5*^, Gamil Gamal Hasan ^6^, Mohammed Althamthami ^2^, Sabrina Ziad ^4^, Tarik Otmane ^3^, David W. Rooney ^5^

^1^ Laboratory of LARGHYDE, University of Biskra, P.O. Box 145, Biskra 07000, Algeria.

^2^Department of Industrial Chemistry, University of Biskra, P.O. Box 145, Biskra 07000,

Algeria.

^3^Scientific and Technical Research Center for Arid Zones CRSTRA, University of Biskra, PO Box 145, Biskra 07000, Algeria.

^4^Laboratory of LARHYSS, University of Biskra, BP 145 RP, Biskra 07000, Algeria.

^5^School of Chemistry and Chemical Engineering, Queen’s University Belfast, Belfast BT9 5AG, Northern Ireland, UK.

^6^Department of Process Engineering and Petrochemical, Faculty of Technology, University of El Oued, El Oued 39000, Algeria

*Corresponding author: Ahmed I. Osman ([aosmanahmed01@qub.ac.uk](mailto:aosmanahmed01@qub.ac.uk))

**Mechanism of ZnO Formation in ZnO@AC Composite**

During the impregnation of DPS with a zinc chloride (ZnCl_2_) solution (, ZnCl_2_ is adsorbed onto the surface of the carbon precursor. As the temperature increases (up to the melting temperature of ZnCl_2_) during pyrolysis, ZnCl_2_ acts as a dehydrating agent and removes hydrogen (H) and oxygen (O) atoms from the surface functional groups of the carbon material. This reaction releases water vapor (H_2_O) as a byproduct. The released H_2_O then reacts with ZnCl_2_ to form zinc oxide chloride hydrate (Zn_2_OCl_2_.2H_2_O), through **Eq. S.1** as reported by (Ma 2017). At the high pyrolysis temperature, this intermediate compound decomposes through **Eq. S.2** (Ma 2017), releasing ZnCl_2_ gas and forming ZnO particles. The volatile gas diffuses within the porous structure of the carbonaceous material. This diffusion process contributes to developing additional porosity within the carbon material. Finally, the ZnO particles formed during the decomposition remain embedded within the carbon matrix, resulting in the ZnO@AC composite.

$\left( C_{x}H_{y}O_{z} \right)+2Zn{Cl}_{2} \underset{\to}{\Delta} {\left( C_{x}H_{y-6}O_{z-3} \right) + Zn}_{2}O{Cl}_{2}.2H_{2}O+2HCl$ (S.1)

${Zn}_{2}O{Cl}_{2}.2H_{2}O \underset{\to}{\Delta} ZnO+{Zn{Cl}_{2}}_{(g)}+2H_{2}O$ (S.2)


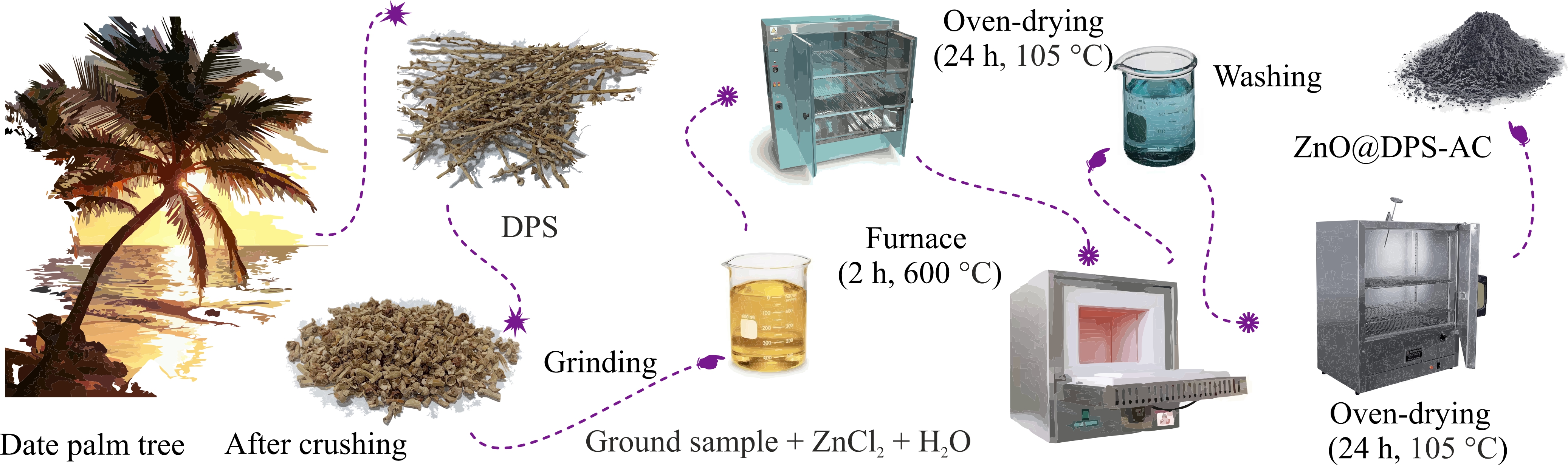


**Fig. S1**. Schematic representation of ZnO@DPS-AC composite preparation.

**
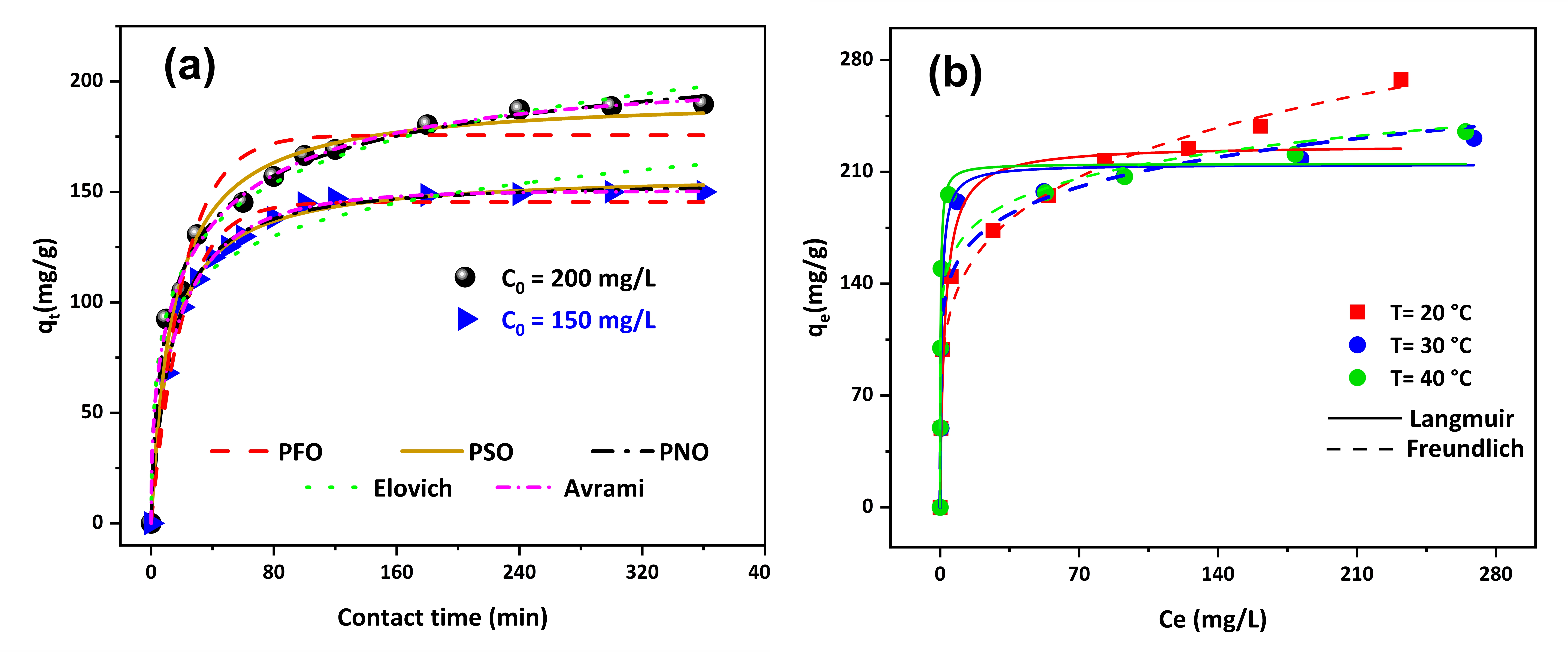
**

**Fig. S2**. (**a**) Kinetics modeling, and (**b**) Isotherm modeling of MO removal by ZnO@DPS-AC composite.





**Fig. S3**. Linear fitting of Intra-Particle Diffusion model.

**Table S1**. Kinetic and isotherm models used in this study.

| **Model** | **Equation** | **Eq.** | **Parameters** | **Referance** |
| --- | --- | --- | --- | --- |
| Pseudo-First-Order (PFO) | $q_{t}=q_{e}(1-{exp}^{(-k_{1}t)})$ | (S.3) | **q_e_**, **q_t_** (mg/g): adsorption capacity of MO at equilibrium and at time t, respectively. **k_1_** (1/min): Rate constant of Pseudo-First-Order. | (Lagergren 1898) |
| Pseudo-Second-Order (PSO) | $q_{t}=\frac{{q_{e}}^{2}k_{2}t}{1+q_{e}k_{2}t}$ | (S.4) | **k_2_** (g /mg.min): Pseudo-Second-Order rate constant. | (Blanchard et al. 1984) |
| Avrami | $q_{t}=q_{e}(1-{exp}^{-{{(k}_{AV} t)}^{n_{AV}}}$ | (S.5) | **k_AV_** (1/min): Avrami kinetic constant. **n_AV_**: Fractional adsorption order of Avarmi. | (Avrami 1939) |
| Elovich | $q_{t}=\frac{1}{\beta}ln(1+\alpha\beta t)$ | (S.6) | **β** related to the extent of surface coverage.  **α** (mg/g.min): Initial adsorption rate. | (Roginsky &Zeldovich 1934) |
| Intra-Particle Diffusion (IPD) | $q_{t}=k_{IPD}\sqrt{t}+C$ | (S.7) | **k_IPD_** (mg/ [g. min^0.5^]): Intraparticle diffusion rate constant. **C** (mg/g): Constant related to the thickness of the boundary layer. | (Weber Jr &Morris 1963) |
| Langmuir | $q_{e}=\frac{q_{m}K_{L}C_{e}}{1+K_{L}C_{e}}$ | (S.8) | **q_m_** (mg/g): The maximum saturated monolayer adsorption capacity of PDS-AC adsorbent. **K_L_** (L/mg): The ratio of the adsorption rate and desorption rate.  **C_e_** (mg/L): Concentration of MO at equilibrium. | (Langmuir 1918) |
| Freundlich | $q_{e}=K_{F}C_{e}^{1/n}$ | (S.9) | **K_F_** (mg/g)/(mg/L)^-1/n^ : Freundlich constant.  **n**: Freundlich intensity parameter. | (Freundlich 1907) |

**Table S2**. The typical properties of ZnO@DPS-AC.

| **Property** | **ZnO@DPS-AC** |
| --- | --- |
| Ash content (%) | 65.33 |
| Moisture Content (%) | 2.42 |
| Carbonization Yield (%) | 42.11 |
| Bulk density (g/L) | 508.61 |

**Table S3**. Physical and chemical parameters of ENICAB discharged wastewater before and after ZnO@DPS-AC application.

| **Parameter** | **Initial value** | **After adsorption** | **Standard ^1^** |
| --- | --- | --- | --- |
| Conductivity (µs/cm) | 1272 | 872 | 2800 |
| TDS (mg/L) | 660 | 430 | 1400 |
| TH (mg/L) | 410 | 168 | 500 |
| TAC (mg/L) | 400 | 200 | 65 |
| Dry residue (mg/L) | 1400 | 500 | 2000 |
| Turbidity (NTU) | 840 | 0 | 5 |
| SS (mg/L) | 10 | 0 | 30 |
| BOD (mg/L) | 12 | 0.02 | 30 |
| Salinity (%) | 0.6 | 0.3 | - |
| Ca^2+^ (mg/L) | 64 | 33 | 200 |
| Mg^2+^ (mg/L) | 61 | 21 | 150 |
| NO_2_^-^ (mg/L) | 0.48 | 0.02 | 0.2 |
| NH_4_^+^ (mg/L) | 0.8 | 0.01 | 0.5 |
| HCO_3_^-^(mg/L) | 410 | 205 | 518.6 |
| Cl ^-^ (mg/L) | 165 | 78 | 500 |
| SO_4_^2-^ (mg/L) | 107 | 50 | 400 |
| NO^3-^ (mg/L) | 104 | 16 | 50 |
| Fe^2+^ (mg/L) | 5.44 | 0.02 | 0.3 |
| PO_4_^3-^ (mg/L) | 3.16 | 0.01 | 15 |
| F^-^ (mg/L) | 2.56 | 0 | 1.5 |
| Na^+^ (mg/L) | 87 | 30 | 200 |
| K^+^ (mg/L) | 8 | 0.2 | 12 |
| NH_2_Cl (mg/L) | 0.25 | 0.01 | - |
| Mn (mg/L) | 0.12 | 0.06 | 0.05 |
| Br ^-^ (mg/L) | 0.58 | 0 | - |
| Cu^2+^ (mg/L) | 0.5 | 0 | 2 |
| NH_3_ (mg/L) | 0.17 | 0.03 | - |
| pH | 8.9 | 8.09 | 6.5-9 |

^1^ According to Algerian standards published in the Official Journal of the People's Democratic Republic of Algeria (2012, 2014).

**References**

Avrami M (1939) Kinetics of Phase Change. I General Theory. The Journal of Chemical Physics 7, 1103-1112. <https://doi.org/10.1063/1.1750380>

Blanchard G, Maunaye M, Martin G (1984) Removal of heavy metals from waters by means of natural zeolites. Water Research 18, 1501-1507. <https://doi.org/10.1016/0043-1354(84)90124-6>

Freundlich H (1907) Über die adsorption in lösungen. Zeitschrift für physikalische Chemie 57, 385-470. <https://doi.org/10.1515/zpch-1907-5723>

JORADP (2012) Interministerial order setting the specifications for treated wastewater used for irrigation purposes. ANNEX: Specifications of treated wastewater used for irrigation purposes. Official Journal of People's Democratic Republic of Algeria, pp. 19-20.

JORADP (2014) Executive Decree No. 14-96 relating to the quality of water for human consumption, ANNEX: Human consumption water quality parameters. Official Journal of People's Democratic Republic of Algeria, pp. 15-17.

Lagergren S (1898) About the theory of so-called adsorption of soluble substances. Available: <https://sid.ir/paper/563615/en>

Langmuir I (1918) THE ADSORPTION OF GASES ON PLANE SURFACES OF GLASS, MICA AND PLATINUM. Journal of the American Chemical Society 40, 1361-1403. <https://doi.org/10.1021/ja02242a004>

Ma Y (2017) Comparison of Activated Carbons Prepared from Wheat Straw via ZnCl2 and KOH Activation. Waste and Biomass Valorization 8, 549-559. 10.1007/s12649-016-9640-z

Roginsky S, Zeldovich YB (1934) The catalytic oxidation of carbon monoxide on manganese dioxide. Acta Phys. Chem. USSR 1, 2019. <https://doi.org/10.1021/ja01417a002>

Weber Jr WJ, Morris JC (1963) Kinetics of adsorption on carbon from solution. Journal of the sanitary engineering division 89, 31-59. <https://doi.org/10.1061/JSEDAI.0000430>
